# Association of longitudinal changes in skeletal muscle mass with prognosis and nutritional intake in acutely hospitalized patients with abdominal trauma: a retrospective observational study

**DOI:** 10.3389/fnut.2023.1085124

**Published:** 2023-05-31

**Authors:** Fengchan Xi, Yong You, Weiwei Ding, Tao Gao, Yang Cao, Shanjun Tan, Wenkui Yu

**Affiliations:** ^1^Research Institute of General Surgery, Affiliated Jinling Hospital, Medical School of Nanjing University, Nanjing, Jiangsu, China; ^2^Department of Intensive Care Unit, Women's Hospital of Nanjing Medical University, Nanjing Maternity and Child Health Care Hospital, Nanjing, Jiangsu, China; ^3^Department of Intensive Care Unit, Affiliated Drum Tower Hospital, Medical School of Nanjing University, Nanjing, Jiangsu, China; ^4^Department of General Surgery, Shanghai Clinical Nutrition Research Center, Zhongshan Hospital, Fudan University, Shanghai, China

**Keywords:** skeletal muscle index, nutrition, prognosis, muscle, trauma

## Abstract

**Background:**

The objective of this study was to explore whether longitudinal changes in skeletal muscle mass, from hospital admission to 3  weeks post-trauma, are associated with poor prognosis and nutritional intake in acutely hospitalized patients with abdominal trauma.

**Methods:**

A single-center retrospective observational review was conducted on 103 patients with abdominal trauma admitted to the Affiliated Jinling Hospital, Medical School of Nanjing University between January 2010 and April 2020. Skeletal muscle mass was assessed by abdominal computed tomography (CT) performed within 14 days before surgery and on post-trauma days 1–3 (week 0), 7–10 (week 1), 14–17 (week 2), and 21–24 (week 3). The skeletal muscle index (SMI) at L3, change in SMI per day (ΔSMI/day), and percent change in SMI per day (ΔSMI/day [%]) were calculated. The receiver-operating characteristic (ROC) curve was used to evaluate the discriminatory performance of ΔSMI/day (%) for mortality. Linear correlation analysis was used to evaluate the associations between ΔSMI/day (%) and daily caloric or protein intake.

**Results:**

Among the included patients, there were 91 males and 12 females (mean age ± standard deviation 43.74 ± 15.53 years). ΔSMI_4-1_/d (%) had a ROC-area under the curve of 0.747 (*p* = 0.048) and a cut-off value of −0.032 for overall mortality. There were significant positive correlations between ΔSMI_4-1_/d (%) and daily caloric intake and protein intake (Y = 0.0007501*X – 1.397, *R*^2^ = 0.282, *R* = 0.531, *p* < 0.001; Y = 0.008183*X - 0.9228, *R*^2^ = 0.194, *R* = 0.440, *p* < 0.001). Δ SMI/day (%) was positively correlated with daily caloric intake ≥80% of resting energy expenditure in weeks 2, 3, and 1–3 post-trauma and with protein intake >1.2 g/kg/d in weeks 3 and 1–3 post-trauma.

**Conclusion:**

Loss of skeletal muscle mass is associated with poor prognosis and nutritional intake in patients admitted to hospital with abdominal trauma.

## 1. Introduction

The prevalence of malnutrition ranges from 7 to 76% in severely injured patients (1). Malnutrition in hospitalized patients has been associated with adverse outcomes, including increased length of hospital stay, morbidity, in-hospital mortality, and cost (1, 2). The Global Leadership Initiative on Malnutrition criteria were established to identify malnutrition in the clinical setting, but there is no gold standard index for diagnosing malnutrition in clinical practice (3). Malnutrition is assessed based on parameters such as body weight, body mass index (BMI), skinfold thickness, and body composition analysis. Accurate diagnosis of malnutrition is affected by post-traumatic edema, serosal effusion, becoming bedridden, and clinician awareness.

Critically ill patients must be provided adequate nutritional support to prevent metabolic deterioration and loss of skeletal muscle mass, which negatively impacts clinical outcomes. Low skeletal muscle mass is an independent predictor of poor clinical prognosis in patients with abdominal trauma (4), and evaluation of the cross-sectional area of the psoas major muscle at the third lumbar vertebral level (L3) has been used as a surrogate measure of decreased strength and functional capacity in older adults (2, 3, 5, 6). Computed tomography (CT) assessment of skeletal muscle mass can be used to quantitatively monitor muscle loss. Coupling monitoring of longitudinal changes in skeletal muscle mass with actively supervised nutritional support programs may be beneficial to the prognosis of critically ill or trauma patients.

The objective of the present study was to explore whether longitudinal changes in skeletal muscle mass, from hospital admission to 4 weeks post-trauma, are associated with poor prognosis and nutritional intake in acutely hospitalized patients with abdominal trauma. Our hypothesis is that longitudinal changes in skeletal muscle are associated with prognosis in trauma patients, and we then test this hypothesis using the receiver-operating characteristic (ROC) curves. Furthermore, we confirm the correlation between nutrition intake and longitudinal changes in skeletal muscle.

## 2. Methods

This study was performed at the Research Institute of General Surgery, Affiliated Jinling Hospital, Medical School of Nanjing University. The protocol was approved by the Hospital Ethics Review Board on November 2, 2021 (Approval #: 2021NZKY-045-01). Our unit is a national trauma center, it is also an abdominal trauma center, with 46 ICU beds, and about 200 trauma patients are admitted every year, and about 150 trauma patients require surgery.

### 2.1. Study design and population

A single-center retrospective observational study included patients with abdominal trauma who admitted to the Affiliated Jinling Hospital, Medical School of Nanjing University between January 2010 and April 2020. Abdominal trauma is defined as blunt or penetrating injury to the abdominal cavity. The abdominal cavity upper limit extends from the horizontal plane passing through the base of the xiphoid process and the spinous process of the 12th dorsal vertebra. It is situated between the cephalad side of the thoracic cavity and the caudal side of the pelvis. The pubic symphysis marks the beginning of the lower boundary of the abdominal cavity, which continues along the entire inguinal arc and iliac crest, and terminates at the spinous process of the 5th lumbar vertebra (7). Inclusion criteria were: (1) age 18–80 years; (2) length of hospital stay >30 days; (3) abdominal CT performed within 14 days before surgery and on post-trauma days 1–3 (week 0), 7–10 (week 1), 14–17 (week 2), and 21–24 (week 3). Exclusion criteria were: (1) missing data; (2) discharge within 72 h of hospitalization; (3) pregnancy; (4) history of mental illness.

### 2.2. Data collection

The medical records of included patients were retrospectively reviewed. Baseline demographic and clinical characteristics were recorded at hospital admission, including sex, age, body weight, BMI (ratio of body weight [kg] to height [m^2^]), trauma type, vital signs at arrival, injured organ, readmission, abbreviated injury scale (AIS), injury severity score (ISS), route of nutrition, nutritional risk screening (NRS), serum levels of albumin, and mechanical ventilation, vasopressor support and transfusion. Clinical chemistry included leukocyte count, C-reactive protein (CRP), procalcitonin (PCT), albumin, and transferrin. The age-adjusted Charlson comorbidity index (ACCI) was used to evaluate comorbidity.

Skeletal muscle mass was assessed by abdominal CT. The skeletal muscle index (SMI) at L3 was calculated as skeletal muscle area (SMA) (cm^2^)/height (m^2^) at hospital admission and post-trauma week 0, week 1, week 2, and week 3. Skeletal muscle was demarcated using predetermined thresholds: −29 to +150 Hounsfield units (HU) for muscle tissue, −150 to −50 HU for visceral adipose tissue, and − 190 to −30 HU for subcutaneous and intramuscular adipose tissue (Neusoft, China). Cut-off values of the SMI for low skeletal muscle mass were 42.08 cm^2^/m^2^ for men and 37.35 cm^2^/m^2^ for women, according to our previously published study (4).

Clinical outcomes included 30-day mortality, 60-day mortality, 90-day mortality, length of hospital stay, hospital costs, and use of mechanical ventilation, continuous renal replacement therapy (CRRT), vasopressors, transfusion and/or laparotomy.

### 2.3. Definitions

Abdominal CT performed during post-trauma weeks 0–3 was denoted as CT1, CT2, CT3, and CT4, respectively.

**Figure 1:**
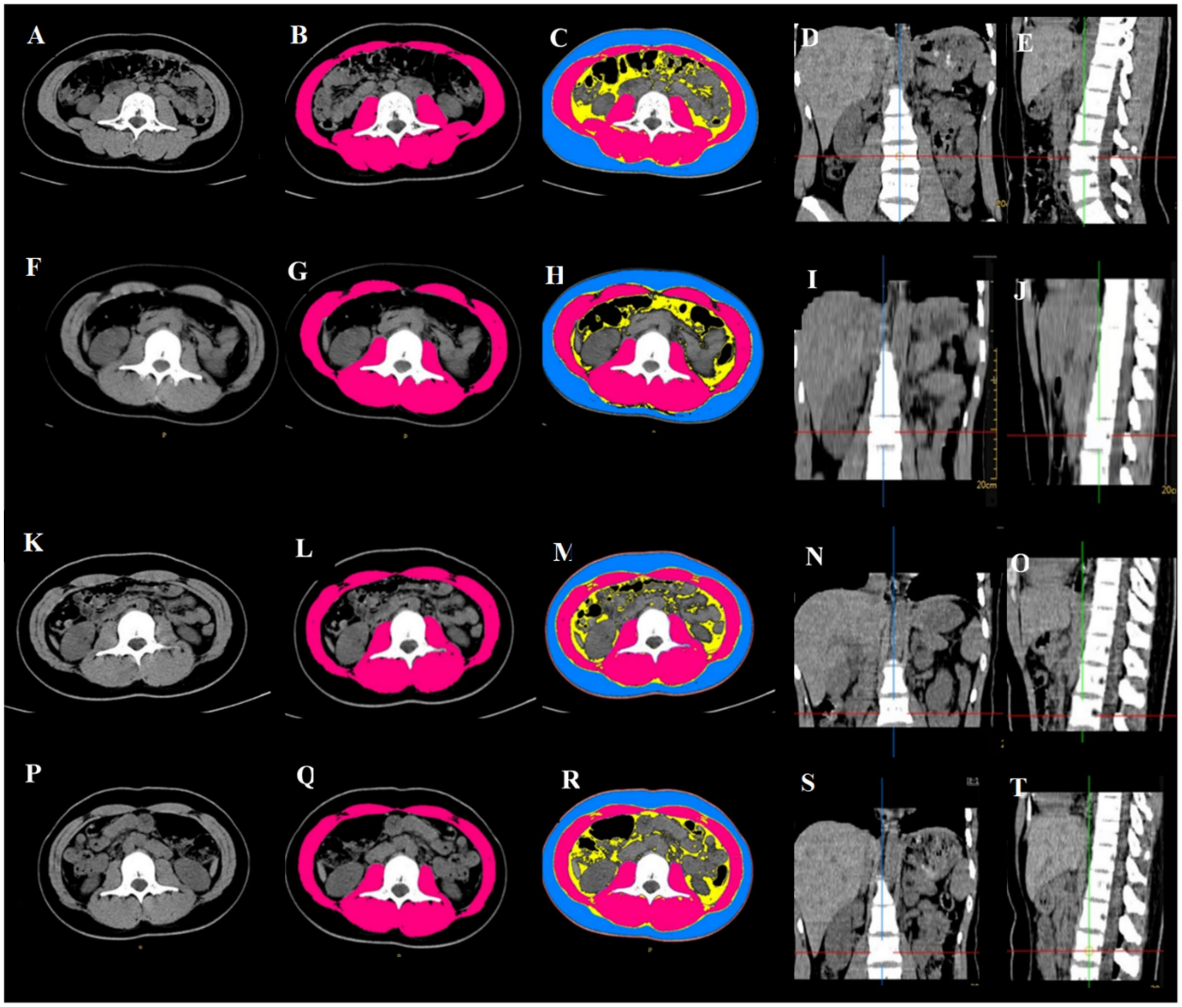
Abdominal CT scan showing changes in skeletal muscle mass at L3 after abdominal trauma in an 18-year female. **(A–E)** Within 14 days before surgery: SMA = 104 cm^2^, SMI = 38.81 cm^2^/m^2^; **(F–J)** post-trauma week 1: SMA = 83.5 cm^2^, SMI = 31.42 cm^2^/m^2^; **(K–O)** post-trauma week 2: SMA = 81.22 cm^2^, SMI = 31.42 cm^2^/m^2^; **(P–T)** post-trauma week 3: SMA = 78.03 cm^2^, SMI = 29.36 cm^2^/m^2^. **(A,F,K,P)** horizontal cross section at L3; **(B,G,L,Q)**: skeletal muscle mass at L3 (red); **(C,H,M,R)** subcutaneous fat (blue), abdominal fat (yellow), skeletal muscle (red); **(D,I,N,S)** coronal plane, L3 is at the intersection of the red line and blue line; **(E,J,O,T)** saggital plane, L3 is at the intersection of the red line and green line.

Skeletal muscle area and SMI at L3 measured on abdominal CT performed during post-trauma weeks 0–3 were denoted as SMA_1_, SMA_2_, SMA_3_ and SMA_4_ and SMI_1_, SMI_2_, SMI_3_ and SMI_4_, respectively (Figure 1).

Changes in SMA or SMI were calculated as ΔSMA_2-1_ = SMA_2_-SMA_1_, ΔSMA_3-2_ = SMA_3_-SMA_2_, ΔSMA_4-3_ = SMA_4_-SMA_3_ and ΔSMA_4-1_ = SMA_4_-SMA_1_, or ΔSMI_2-1_ = SMI_2_-SMI_1_, ΔSMI_3-2_ = SMI_3_-SMI_2_, ΔSMI_4-3_ = SMI_4_-SMA_3_ and ΔSMI_4-1_ = SMI_4_-SMI_1_.

Change in SMA or SMI per day was calculated as ΔSMA = (post SMA – pre SMA)/ days between initial abdominal CT scan and final abdominal CT scan, or ΔSMI = (post SMI – pre SMI)/ days between initial abdominal CT scan and final abdominal CT scan.

Percent change in SMA (ΔSMA/day [%]) or SMI (ΔSMI/day [%]) per day was calculated as ΔSMA/day (%) = (SMA at final abdominal CT scan – SMA at initial abdominal CT scan)/SMA at initial abdominal CT scan × 100/days between initial abdominal CT scan and final abdominal CT scan or ΔSMI/day (%) = (SMI at final abdominal CT scan – SMI at initial abdominal CT scan)/SMI at initial abdominal CT scan × 100/days between initial abdominal CT scan and final abdominal CT scan. This approach was adapted from previous studies that have calculated Δ SMI/year (%) (8, 9) and Δ SMI/ month (%) (10).

### 2.4. Nutritional therapy and measurement

Patients with abdominal trauma were provided clinical nutrition according to ESPEN guidelines. Low-calorie nutrition (not exceeding 70% of resting energy expenditure) was provided in the acute phase. The caloric intake was increased to 80–100% of resting energy expenditure and protein intake was 1.2–2.0 g /kg/d after 72 h.

### 2.5. Statistics

Statistical analyses were conducted using SPSS version 22 software (IBM, Inc., Armonk, NY, United States). Data are presented as mean ± SD or median and interquartile ranges for continuous variables and were compared using the *t*-test or Mann–Whitney *U* test, as appropriate. Categorical data are expressed as numbers and proportions and were compared using the χ^2^ test or Fisher’s exact test, as appropriate. Nonparametric tests, such as the Mann–Whitney *U* test or Wilcoxon test, were used for non-normally distributed data. Images were evaluated by three radiologists. We used Kendall’s W consistency coefficient and intraclass correlation coefficients (ICC) to test the consistency of CT images and assess their quality. Kendall’s *W* was 0.834 (*p* < 0.001) and the ICC was 0.801 (95% confidence interval [CI], 0.758–0.829) (*p* < 0.001) (zero indicates no agreement between raters; 1 indicates perfect agreement), indicating a strong consistency between the three clinicians. The ROC curve was used to evaluate the discriminatory performance of ΔSMI/day (%) for mortality (area under the curve [AUC] > 0.9 high accuracy; 0.7–0.9 moderate accuracy; 0.5–0.7 low accuracy). Youden’s index was used to determine the optimal cut-off value. Linear correlation analysis was used to evaluate the associations between ΔSMI/day (%) and daily caloric intake (caloric intake ≥80% of resting energy expenditure, 50–80% of resting energy expenditure, < 50% of resting energy expenditure) (11), and between ΔSMI/day (%) and daily protein intake (protein intake ≥2.0 g/kg/d, 1.2–2.0 g/kg/d, < 1.2 g/kg/d). *p* < 0.05 was considered statistically significant.

## 3. Results

### 3.1. Study population

**Figure 2:**
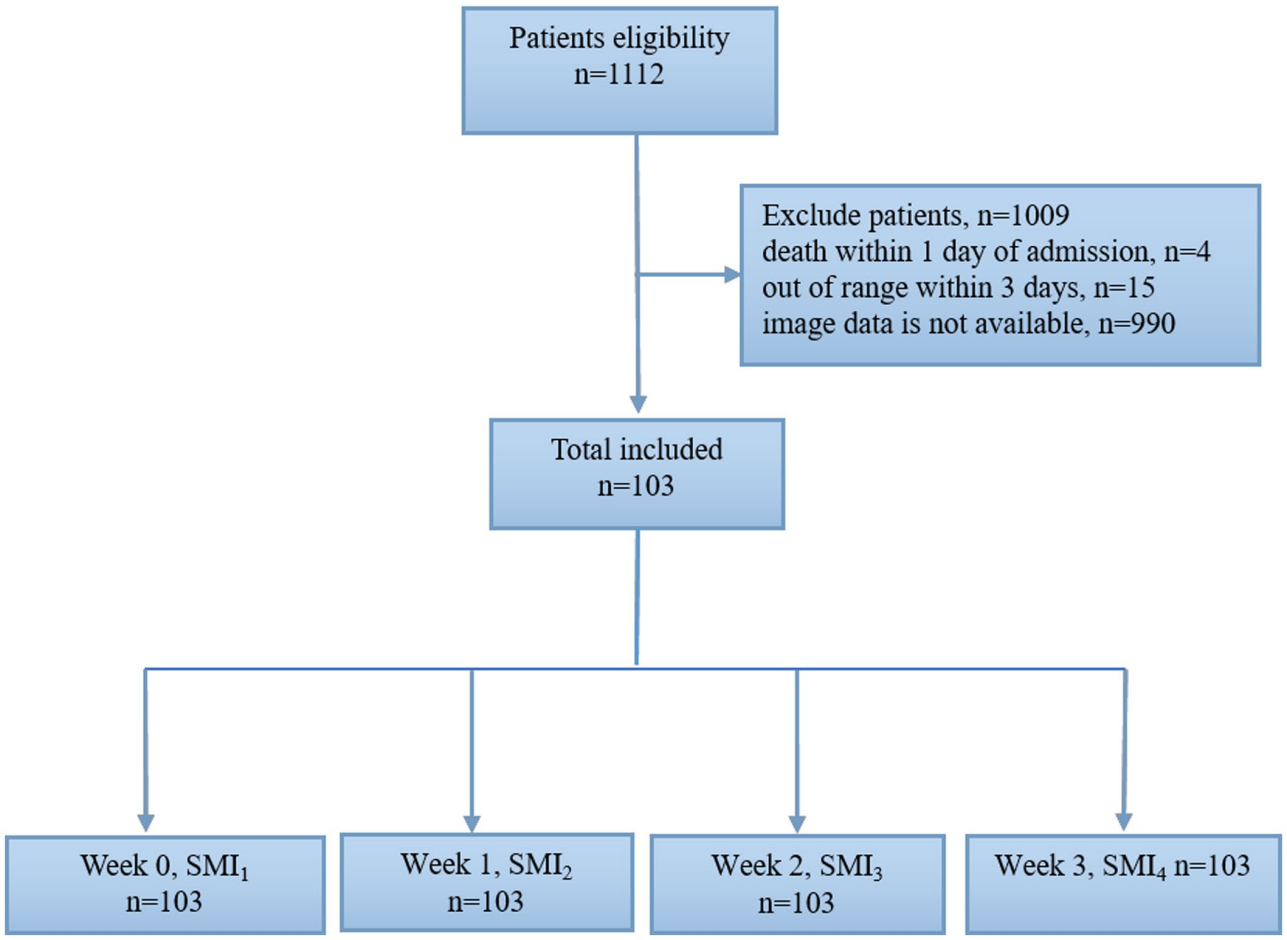
Study flow chart.

A flow chart of the study participants is shown in Figure 2. A total of 1,112 patients with abdominal trauma were admitted to the Affiliated Jinling Hospital, Medical School of Nanjing University between January 2010 and April 2020. Of these, 990 patients had missing data, 15 patients were discharged within 3 days of admission, and 4 patients died within 1 day of admission. Finally, 103 patients were included in the analysis. The demographic and clinical characteristics of the included patients were summarized in Table 1. 88.3% (*n* = 91) of patients were males. 99 (96%) patients suffered blunt abdominal trauma, 4 (4%) patients suffered penetrating trauma, and 18 (17.5%) patients were readmissions.

**Table 1:** Demographic and clinical characteristics of the included patients.

Characteristics	Value
Age, y, Mean ± SD	43.74 ± 15.53
*Sex, n (%)*	
Male	91 (88.3)
Female	12 (11.7)
Weight, kg, Mean ± SD	66.89 ± 11.58
BMI, kg/m^2^, Mean ± SD	22.85 ± 3.36
ACCI score, Median (IQR)	1 (0–2)
*Type of injury, n (%)*	
Blunt	99 (0.96)
Penetrating	4 (0.04)
*Vital signs at arrival, Median (IQR)*	
GCS	14 (13–15)
HR	82 (72–98)
RR	17 (15–20)
SBP	123 (109–130)
*Injured organs, n (%)*	
Gastric	1 (0.9)
Duodenal	2 (1.8)
Small intestine	16 (15.5)
Colon	3(2.9)
Mesentery	4 (3.9)
Liver	15 (14.6)
Spleen	6 (5.8)
Pancreas	6 (5.8)
Multiple abdominal Injuries	50 (48.5)
Readmission, *n* (%)	18 (17.5)
*ISS, n (%)*	
15–24	27 (26.2)
25–34	33 (32.0)
35–44	6 (5.8)
≥45	5 (4.8)
ISS ≥ 16, *n* (%)	71 (68.9)
AIS HEAD score ≥ 3, *n* (%)	18 (17.5)
AIS THORAX score ≥ 3, *n* (%)	52 (50.5)
AIS ABDOMEN score ≥ 3, *n* (%)	77 (74.7)
AIS EXTREMITIES score ≥ 3, *n* (%)	16 (15.5)
*Route of nutrition, n (%)*	
Enteral nutrition	9 (0.0)
Parenteral nutrition	19 (24.0)
Combined enteral and parenteral nutrition	59 (60.0)
None	16 (16.0)
Leukocyte (◊10^9^/L), Mean ± SD	14.02 ± 6.73
CRP (mg/L), Mean ± SD	27.76 ± 12.43
PCT (Mean ± SD)(ng/mL), Mean ± SD	3.53 ± 0.77
NSR 2002, Mean ± SD	3.49 ± 1.67
Albumin (g/L), Mean ± SD	31.3 ± 5.66
Transferrin (g/L), Mean ± SD	1.76 ± 0.54

SD, standard deviation; BMI, body mass index; ACCI, age-adjusted Charlson comorbidity index; GCS, Glasgow coma score; SBP, systolic blood pressure; RR, respiratory rate; ISS, injury severity score; AIS, abbreviated injury scale; APACHE-II, acute physiology and chronic health evaluation II; CRP, C-reactive protein; PCT, procalcitonin; NRS, nutritional risk screening.

### 3.2. Clinical outcomes

Clinical outcomes of the included patients are summarized in Table 2. Thirty-day, 60-day, and 90-day mortalities were 5.8, 6.8, and 7.8%, respectively. Thirty-nine (37.8%) patients required mechanical ventilation, and the median duration of mechanical ventilation was 18 days. Twelve (11.6%) patients required CRRT, and the median duration of CRRT was 7 days. Twenty-one (20.2%) patients required norepinephrine. Forty-one (39.8%) patients received blood transfusion, and 53 (51.4%) patients underwent laparotomy.

**Table 2:** Clinical outcomes of the included patients.

Clinical outcomes	Value
30-day mortality, *n* (%)	6 (5.8)
60-day mortality, *n* (%)	7 (6.8)
90-day mortality, *n* (%)	8 (7.8)
Hospital LOS, d, Mean ± SD	35.46 ± 7.42
Hospital cost, ×10^4^$, Mean ± SD	2.54 ± 1.42
Mechanical ventilation, *n* (%)	39 (37.8)
Mechanical ventilation, d, Median (IQR)	18 (6–38)
CRRT, *n* (%)	12 (11.6)
CRRT, d, Median (IQR)	7 (3–17)
Vasopressor support, *n* (%)	21 (20.3)
Transfusion, *n* (%)	41 (39.8)
Transfusion volume, mL, Median (IQR)	1,535 (875–3,800)
Massive transfusion, >10 RBC units, *n* (%)	15 (14.6)
Laparotomy, *n* (%)	53 (51.4)

LOS, length of stay; CRRT, continuous renal replacement therapy; IQR, interquartile range.

### 3.3. Skeletal muscle area and skeletal muscle index at L3

#### 3.3.1. Skeletal muscle area and skeletal muscle index

**Figure 3:**
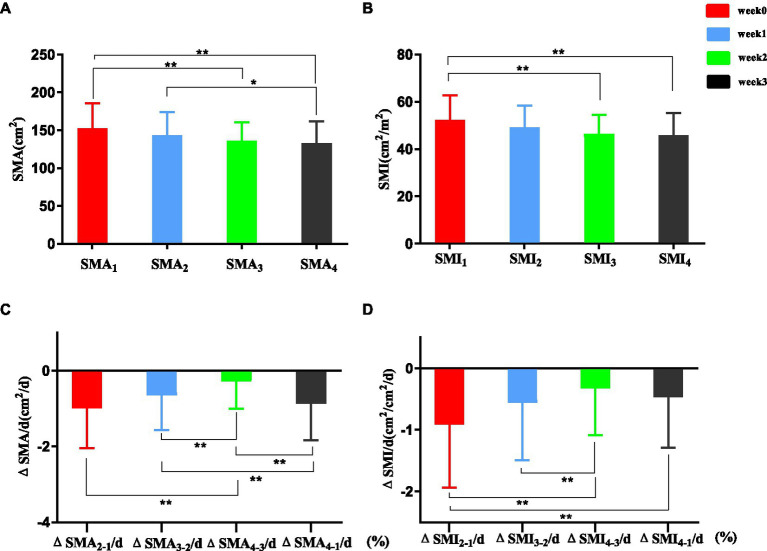
SMA **(A,C)** and SMI **(B,D)** at L3. Numbers are mean ± SEM (*n* = 103). *p < 0.05, **p < 0.01.

Skeletal muscle area at L3 during post-trauma weeks 0–3 is shown in Figure 3A. SMA_3_ and SMA_4_ were significantly lower than SMA_1_ (135.78 ± 24.83 vs. 152.63 ± 32.72 cm^2^, *p* < 0.001; 132.83 ± 28.42 vs. 152.63 ± 32.72 cm^2^, *p* < 0.001). SMA_4_ was significantly lower than SMA_2_ (132.83 ± 28.42 vs. 143.73 ± 30.42 cm^2^, *p* = 0.038). SMI at L3 during post-trauma weeks 0–3 is shown in Figure 3B. SMI_3_ and SMI_4_ were significantly lower than SMI_1_ (46.47 ± 7.92 vs. 52.33 ± 10.28 cm^2^/m^2^, *p* < 0.001, 45.90 ± 9.25 vs. 52.33 ± 10.28 cm^2^/m^2^, *p* < 0.001).

#### 3.3.2. ΔSMA/d (%) and ΔSMI/d (%)

ΔSMA/d (%) at L3 is shown in Figure 3C. ΔSMA_2-1_/d (%) showed a significantly greater decrease than ΔSMA_4-3_/d (%) (median [IQR] –0.99 [−2.05–0.11] vs. –0.28 [−1.00–0.18], *p* < 0.01). ΔSMA_3-2_/d (%) was significantly greater than ΔSMA_4-3_/d (%) (median [IQR] –0.65 [−1.57 to 0.00] vs. –0.28 [−1.00–0.18], *p* < 0.01). ΔSMA_4-1_/d (%) was significantly greater than ΔSMA_3-2_/d (%) (median [IQR] –0.87 [−1.84 to 0.26] vs. –0.65 [−1.57 to 0.00], *p* < 0.01) and ΔSMA_4-3_/d (%) (median [IQR] -0.87 [−1.84 to 0.26] vs. −0.28 [−1.00 to 0.18], *p* < 0.01).

ΔSMI/d (%) is shown in Figure 3D. ΔSMI_2-1_/ d (%) showed a significantly greater decrease than ΔSMI_4-3_/d (%) and ΔSMI_4-1_/d (%) (median [IQR] –0.91 [−1.94–0.07] vs. –0.33 [−1.09–0.03], *p* < 0.01; −0.91 [−1.94–0.07] vs. –0.47 [−0.89–0.00], *p* < 0.01).

### 3.4. ΔSMI/d (%) as a prognostic predictor

**Figure 4:**
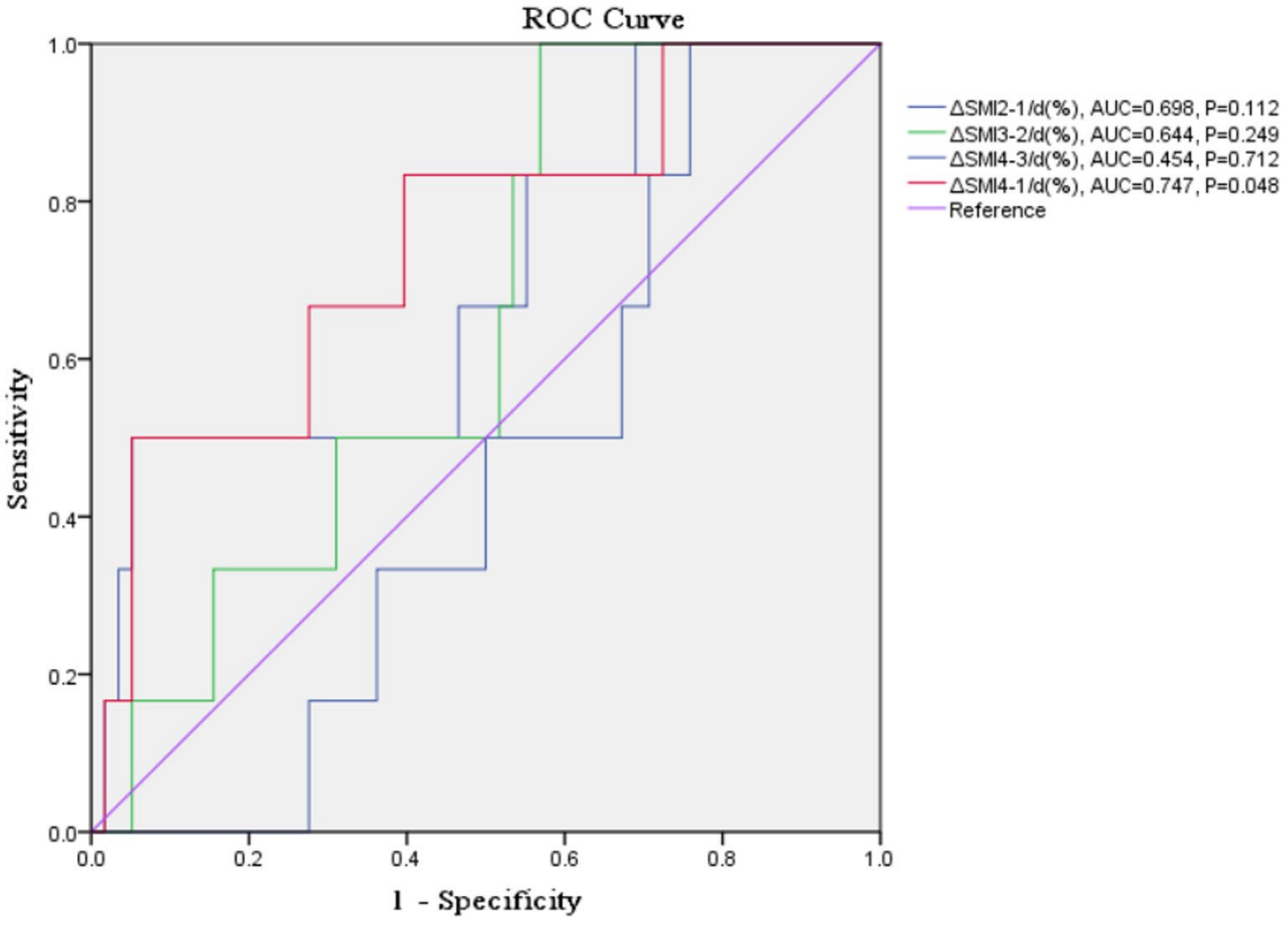
Receiver-operating characteristic (ROC) curve of ΔSMI/d (%) in predicting mortality.

Receiver-operating characteristic curve analysis examining the predictive value of Δ SMI_4-1_/d (%) for overall mortality is shown in Table 3 and Figure 4. Δ SMI_4-1_/d (%) had a ROC-AUC of 0.747 (*p* = 0.048) and a cut-off value of −0.032 (Table 3).

**Table 3:** Receiver-operating characteristic (ROC) analysis examining the predictive value of ΔSMI/d (%) for mortality.

	AUC	95%CI value of *p*	Sensitivity		Specificity	Youden’s Index	Cut-off
ΔSMI_3-2_/d (%)	0.644	(0.459, 0.828)	0.249	100.0%	43.1%	0.431	−0.389
ΔSMI_2-1_/d (%)	0.698	(0.467, 0.930)	0.112	5.0%	94.8%	0.448	0.469
ΔSMI_4-3_/d (%)	0.454	(0.277, 0.631)	0.712	100.0%	24.1%	0.241	−0.599
ΔSMI_4-1_/d (%)	0.747	(0.535, 0.959)	0.048*	50.0%	94.8%	0.448	−0.032

AUC, area under the curve. *p < 0.05.

### 3.5. Nutritional therapy

Daily caloric intake were 641.27 ± 335.02 kcal/d, 1274.76 ± 686.77 kcal/d, 1334.39 ± 815.83 kcal/d, 1212.02 ± 489.70 kcal/d, respectively in post-trauma week 1, week 2, week 3, week 1–3. Daily protein intake were 17.35 ± 8.97 g/d, 47.38 ± 24.83 g/d, 66.86 ± 28.34 g/d, 44.36 ± 29.63 g/d, respectively in post-trauma week 1, week 2, week 3, week 1–3.

### 3.6. ΔSMI/d (%) and daily caloric intake and protein intake

**Figure 5:**
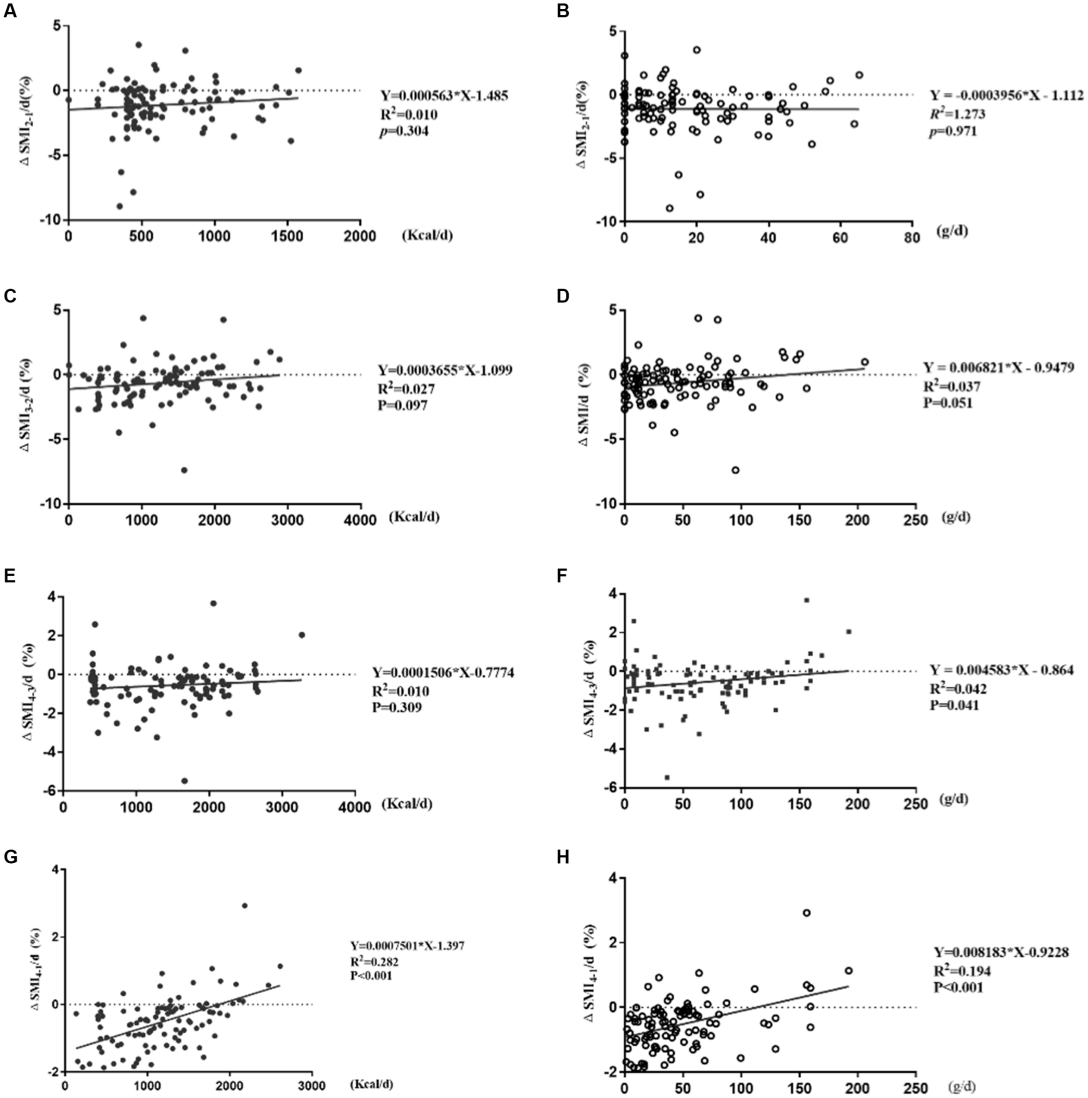
Correlation analysis of ΔSMI/d (%) and daily caloric and protein intake. **(A,B)** Correlation of ΔSMI_2-1_/d (%) and caloric and protein intake; **(C,D)** correlation of ΔSMI_3-2_/d (%) and caloric and protein intake; **(E,F)** correlation of ΔSMI_4-3_/d (%) and caloric and protein intake; **(G,H)** correlation of ΔSMI_4-1_/d (%) and caloric and protein intake.

ΔSMI_2-1_/d (%), ΔSMI_3-2_/d (%), ΔSMI_4-3_/d (%) were not correlated with daily caloric intake, and also ΔSMI_2-1_/d (%), ΔSMI_3-2_/d (%) with protein intake (*p* > 0.05). But there were significant positive correlations between ΔSMI_4-3_/d (%) and daily protein intake (*Y* = 0.004583*X – 0.864, *R*^2^ = 0.042, *R* = 0.205, *p* = 0.041), ΔSMI_4-1_/d (%) and daily caloric intake and protein intake (Y = 0.0007501*X – 1.397, *R*^2^ = 0.282, *R* = 0.531, *p* < 0.001; *Y* = 0.008183*X – 0.9228, *R*^2^ = 0.194, *R* = 0.440, *p* < 0.001; Figure 5).

**Figure 6:**
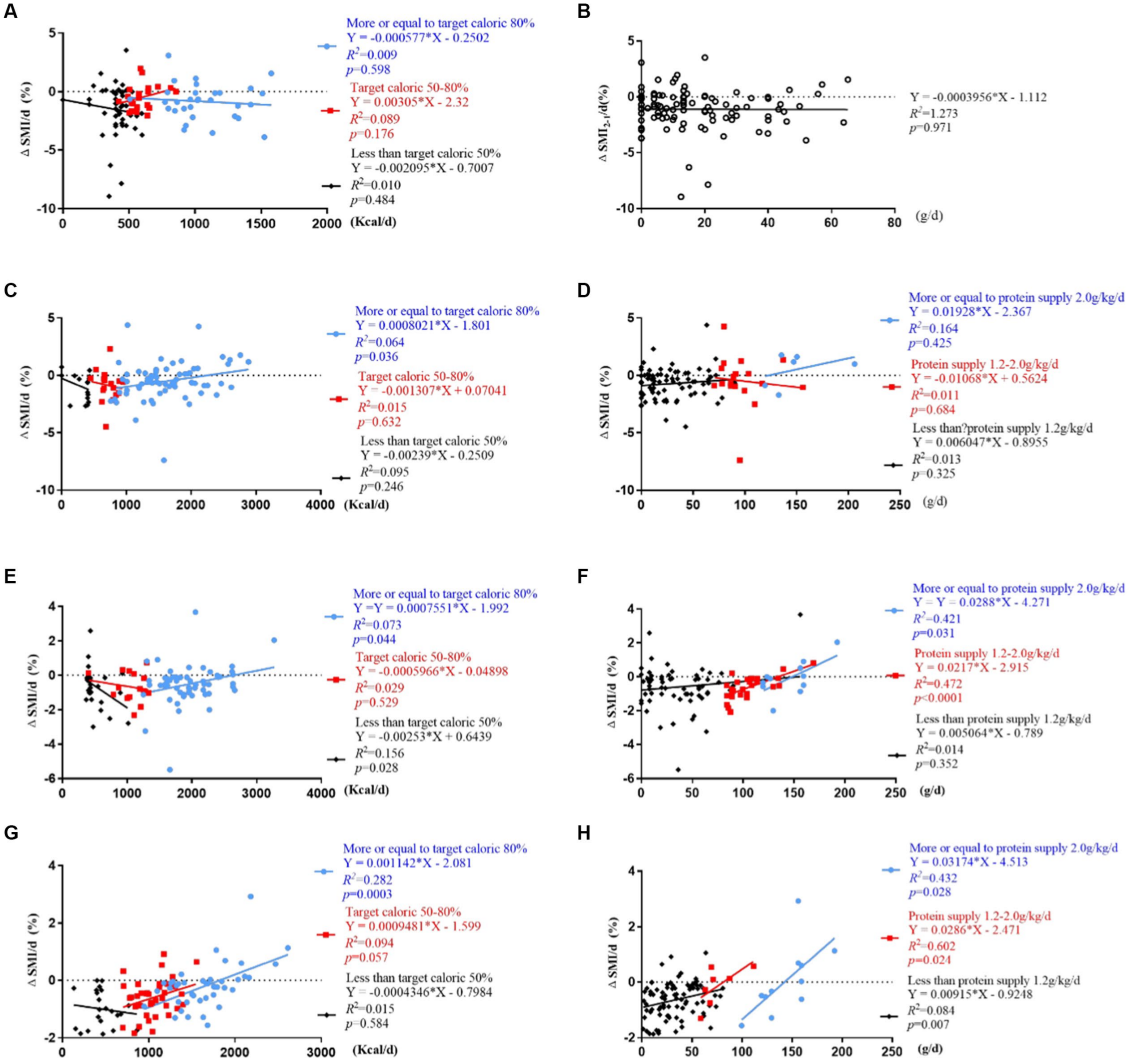
Correlation analysis of ΔSMI/d (%) and daily caloric and protein intake stratified by caloric (<50%, 50–80% ≥ 80% of resting energy expenditure) and protein (<1.2 g/kg/d, 1.2–2 g/kg/d, ≥ 2 g/kg/d) target levels. **(A,B)** Correlation of ΔSMI_2-1_/d (%) and caloric and protein intake; **(C,D)** correlation of ΔSMI_3-2_/d (%) and caloric and protein intake; **(E,F)** correlation of ΔSMI_4-3_/d (%) and caloric and protein intake; **(G,H)** correlation of ΔSMI_4-1_/d (%) and caloric and protein intake.

ΔSMI_2-1_/d (%) were not correlated with daily caloric intake ≥80%, 50–80%, or <50% of resting energy expenditure and protein intake (*p* > 0.05) (Figures 6A,B).

ΔSMI_3-2_/d (%) was positively correlated with daily caloric intake ≥80% of resting energy expenditure (*Y* = 0.0008021*X – 1.801, *R*^2^ = 0.064, *R* = 0.253, *p* = 0.036). It was not correlated with daily protein intake (*p* > 0.05) (Figures 6C,D).

ΔSMI_4-3_/d (%) were positively correlated with daily caloric intake ≥80% of resting energy expenditure (*Y* = 0.0007551*X – 1.992, *R*^2^ = 0.073, *R* = 0.270, *p* = 0.044) and protein intake >1.2 g/kg/d (*Y* = 0.0217*X – 2.951, *R*^2^ = 0.472, *R* = 0.687, *p* < 0.001; *Y* = 0.0288*X – 4.271, *R*^2^ = 0.421, *R* = 0.649, *p* = 0.031), and negatively correlated with daily caloric intake <50% of resting energy expenditure (*Y* = −0.00253*X + 0.6439, *R*^2^ = 0.156, *R* = 0.395, *p* = 0.028) (Figures 6E,F).

ΔSMI_4-1_/d (%) were positively correlated with daily caloric intake ≥80% of resting energy expenditure (*Y* = 0.001142*X – 2.081, *R*^2^ = 0.282, *R* = 0.531, *p* < 0.001) (Figure 6G), and protein intake <1.2 g/kg/d, 1.2–2.0 g/kg/d and ≥2.0 g/kg/d (*Y* = 0.00915*X-09248, *R*^2^ = 0.084, *R* = 0.289, *p* = 0.007; *Y* = 0.0286*X – 2.471, *R*^2^ = 0.602, *R* = 0.775, *p =* 0.024; *Y* = 0.03174*X – 4.513, *R*^2^ = 0.432, *R* = 0.657, *p* = 0.028) (Figure 6H).

**Figure 7:**
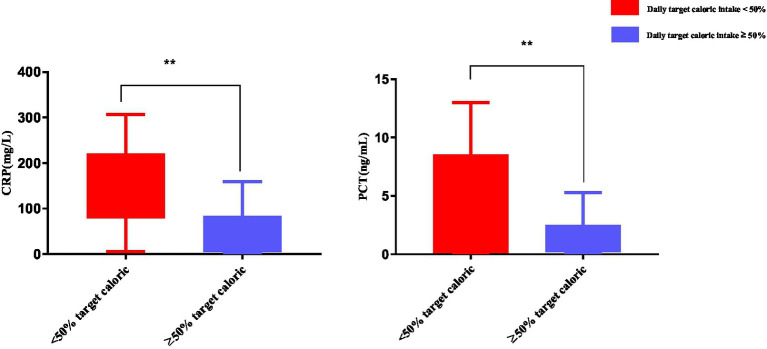
Inflammatory response in patients with a daily caloric intake <50% of resting energy expenditure. Numbers are mean ± SEM (*n* = 103). **p < 0.01.

The inflammatory response was evaluated in patients with a daily caloric intake <50% (*n* = 29) or ≥50% (*n* = 74) of resting energy expenditure. Findings showed that CRP and PCT levels were significantly higher in patients with a daily caloric intake <50% compared to ≥50% of resting energy expenditure (*p* < 0.01) (Figure 7).

## 4. Discussion

This study explored whether longitudinal changes in skeletal muscle mass, from hospital admission to 3 weeks post-trauma, are associated with poor prognosis and nutritional intake in acutely hospitalized patients with abdominal trauma. Findings showed a rapid decrease in skeletal muscle mass at L3 in week 1 post-trauma, and ΔSMI_4-1_/d (%) was capable of predicting poor prognosis. ΔSMI/day (%) was an effective indicator of nutritional status, and increasing daily caloric intake to ≥80% of resting energy expenditure at 2 weeks post-trauma and daily protein intake to ≥1.2 g/kg/d at 3 weeks post-trauma slowed depletion of skeletal muscle mass at L3. These results suggest skeletal muscle mass should be evaluated in the first week for abdominal trauma patients.

Malnutrition is an independent risk factor for complications, mortality, long hospital length of stay, and reduced quality of life in critically ill and trauma patients (1, 12, 13). Common indicators of malnutrition include weight, BMI, skin fold thickness, and body composition analysis; however, these indicators are highly variable and may not accurately represent nutritional status in all situations (14, 15). Weight and height are often determined from a subjective estimation in critically ill patients (16). Infection, bleeding, and edema affect body mass and body composition analysis. Severe stress and hypercatabolism during early trauma may affect resting energy consumption and a patient’s metabolic rate. Currently, there are no standard methods for screening and diagnosing patients with malnutrition. CT evaluation of skeletal muscle is widely used in patients who are critically ill, have undergone surgery, or have cancer (17–19). Evaluation of progressive changes in skeletal muscle mass by CT may provide evidence of a patient’s nutritional status.

In the present study, rates of change in SMA and SMI at L3 varied during the first 3 weeks post-trauma. SMA and SMI were most rapidly depleted in week 1 post-trauma. Accordingly, previous reports have shown that skeletal muscle mass decreases by 2–4% per day in patients in the ICU (20), patients with an ICU length of stay of 40 days can lose up to 40% of total body protein, mostly from skeletal muscle (21), and critically ill patients with intra-abdominal sepsis have an acute and persistent loss of muscle mass averaging an 8% decrease in SMI from baseline at 3 months (22). Skeletal muscle has been identified as a key metabolic and homeostatic organ (23). Muscle plays a central role in protein metabolism when dietary intake fails to meet the body’s protein needs. Skeletal muscle is a reservoir of amino acids and a site where essential amino acids are synthesized. In trauma or other critical conditions, muscle breaks down to supply protein to vital tissues and organs (24). Short-term disuse atrophy (<10 days) is particularly relevant to the depletion of skeletal muscle mass (25). A retrospective study of mechanically ventilated critically ill patients who had an abdominal CT scan (including L3) between day 1 and day 4 after admission to the ICU showed that low SMA was a risk factor for mortality (26). In another study of critically ill patients, muscle loss occurred early and rapidly during the first week of critical illness and was more severe among those with multi-organ failure compared with single organ failure (27). A prospective observational study of patients admitted to the medicine or cardiothoracic ICU with a diagnosis of sepsis or acute respiratory failure showed that skeletal muscle loss in the first 7 days of ICU admission can predict physical function at hospital discharge (28).

Preventative measures that enhance anabolism and reduce catabolism are required to reduce loss of skeletal muscle mass during the acute period of trauma. In the present study, Δ SMI/day (%) was used to investigate the time course of loss of skeletal muscle mass after abdominal trauma. Δ SMI/day (%) was the most severe in the first week (median Δ SMI _2-1_/day (%) = − 0.91%), and Δ SMI/day (%) during 4 weeks post-trauma significantly predicted poor prognosis in our patient population. For comparison, the median Δ SMI/year (%) in cirrhotic patients was estimated as – 0.22% (8).

In the present study, approximately 50% of patients with a daily caloric intake of <50% of resting energy expenditure during week 1 post-trauma appear to consume more caloric and lose more skeletal muscle. These factors may be contributing to their condition, such as severe infection, unstable hemodynamics, serious complications, and co-morbidities. The robust inflammatory response in the acute stage of abdominal trauma in patients with a daily caloric intake of <50% of resting energy expenditure was reflected by higher CRP and PCT levels. Previously studies showed that reaching both protein and energy targets improves survival in critically ill patients (29, 30). A retrospective cohort study of patients hospitalized in an ICU showed underfeeding and overfeeding were harmful to critically ill patients, achieving a caloric target of 70% of resting energy expenditure had a survival advantage, and excessive caloric intake was associated with longer length of stay and length of ventilation (31). A multicenter cohort study of adult patients who were mechanically ventilated for more than 8 days in the ICU showed nutritional intake received during the first week in the ICU was associated with longer survival time and faster physical recovery to 3 months (11). Conversely, two large randomized controlled trials of critically ill patients reported no differences in clinical endpoints after low, normal, or high-calorie intake during early hospitalization in the ICU (32, 33).

Trauma induces complex metabolic changes (34) that involve a neuroendocrine component, release of gastrointestinal hormones, and an inflammatory/immune component. These changes have been associated with anorexia, sepsis, uncontrolled oxidative stress that can damage essential proteins, membrane lipids and DNA, and insulin resistance that can lead to uncontrolled catabolism of peripheral tissues such as fat and muscle (35). Trauma and its management often lead to loss of mobility, which accelerates decrease of skeletal muscle mass and strength.

In the present study, rate of loss of skeletal muscle mass decreased after the caloric target was increased to 80% of resting energy expenditure in post-trauma week 2. At this stage, muscle mass may be maintained by stable hemodynamics, progressive recovery of gastrointestinal function, improved organ function, reduced catabolism, and increased anabolism. Excessive calorie intake may not be associated with changes in skeletal muscle mass; rather, adequate provision of energy and protein is important in reducing muscle loss in critically ill patients (36). A prospective randomized study of patients staying in the ICU ≥ 5 days with outcomes recorded until day 90 showed daily caloric intake of 10–20 kcal/kg and protein intake of 0.8–1.2 g protein/kg, both approaching currently recommended targets, was associated with earlier weaning from invasive mechanical ventilation and longer survival compared to a daily intake above or below these values (37). A prospective, randomized, single-center, pilot clinical trial of mechanically ventilated patients in an adult ICU showed provision of an actively supervised nutritional intervention and providing near target energy requirements was associated with lower hospital mortality (38).

In the present study, ΔSMI/day (%) was positively correlated with daily protein intake during the 4 weeks post-trauma and increasing protein intake to >1.2 g/kg/d at 3 weeks post-trauma slowed depletion of skeletal muscle mass, suggesting that protein supplementation may reduce loss of skeletal muscle mass during the acute period of trauma. Protein is essential for the maintenance of muscle quality and function, and clinical guidelines indicate that critically ill patients may benefit from a protein intake of 1.2 g/kg/d (39). A high-protein intake (1.3 g/kg adjusted body weight/day) is recommended by the 2018 ESPEN guidelines for obese critically ill patients and 2021 ESPEN guidelines for patients with COVID19 (40, 41). In one study of critically ill patients, higher protein delivery in the first week was associated with greater loss of skeletal muscle mass (27). However, further research is needed to optimize recommendations of daily protein intake in acute trauma patients.

However, this study had several limitations. First, the single-center retrospective observational study design introduced the risk of bias and residual confounding. Skeletal muscle changes may be influenced by age, sex, ACCI, severity of trauma, and severity of infection, etc. Further multivariate regression analysis was necessary to eliminate bias due to a number of confounding factors. Second, because the research span is 10 years, the posttraumatic treatment concept may be updated, which may further bias the results. Third, the results were based on short-term follow-up in the hospital, and long-term outcomes were not assessed. Furthermore, the type of abdominal trauma may have influenced the results. Most importantly, nearly 90% of patients are excluded, which may cause bias. In future studies, we will rigorously calculate sample size estimates and develop more scientific inclusion and excluded criteria and follow-up systems. Finally, although this is a single-center, observational, retrospective study in Asia, the methodology can be applied to western populations as well. In the future, a multicenter prospective randomized controlled trial will be required to confirm the impact of nutritional support on abdominal trauma-related skeletal muscle quality. As a follow-up, we will conduct mathematical and computer modeling based on various factors (such as age, height, weight, and disease severity) to develop accurate dose formulas for nutritional supplements. This study will provide a new method for nutritional assessment and a new direction for nutritional treatment.

## 5. Conclusion

This study showed that skeletal muscle mass loss is associated with poor prognosis and nutritional intake in patients admitted to hospital with abdominal trauma. Skeletal muscle mass at L3 decreased rapidly in week 1 post-trauma. ROC curve analysis showed that Δ SMI_4-1_/d (%) was predictive of clinical prognosis. Skeletal muscle changes were correlated with daily caloric and protein intake: there was a positive correlation between Δ SMI/day (%) and daily protein intake in week 3 post-trauma, and a positive correlation between Δ SMI/day (%) and daily caloric and protein intake in weeks 1–3 post-trauma. Δ SMI/day (%) was positively correlated with daily caloric intake ≥80% of resting energy expenditure in weeks 2, 3 and 1–3 post-trauma and with protein intake >1.2 g/kg/d in weeks 3 and 1–3 post-trauma. Evaluation of longitudinal changes in skeletal muscle mass may inform the development of nutritional support programs and improve prognosis in patients admitted to hospital with abdominal trauma.

## Data availability statement

The raw data contributions presented in the study are included in the article/supplementary material, be directed to the corresponding authors.

## Ethics statement

The protocol for this study was reviewed and approved by the Institutional Review Board of the Research Institute of General Surgery, Affiliated Jinling Hospital, Medical School of Nanjing University on November 2, 2021. Written informed consent for participation was not required for this study, according to national and institutional legislation, because it was a retrospective study based on the electronic medical record.

## Author contributions

WY designed the study and performed the data analysis and interpretation. FX, YY, and WD collected the data and wrote the manuscript. ST carried out the study and data analysis. TG and YC helped to draft the tables and figures. WY and ST conceived the study, participated in its design and coordination, and helped draft the manuscript. All the authors critically reviewed the manuscript and approved the final version.

## Funding

This study was supported by the National Major Scientific Research Instruments and Equipments Development Project of National Natural Science Foundation of China (81927808).

## Conflict of interest

The authors declare that the research was conducted in the absence of any commercial or financial relationships that could be construed as a potential conflict of interest.

## Publisher’s note

All claims expressed in this article are solely those of the authors and do not necessarily represent those of their affiliated organizations, or those of the publisher, the editors and the reviewers. Any product that may be evaluated in this article, or claim that may be made by its manufacturer, is not guaranteed or endorsed by the publisher.
